# Tissue distribution of oral vitamin B12 is influenced by B12 status and B12 form: an experimental study in rats

**DOI:** 10.1007/s00394-017-1424-0

**Published:** 2017-03-20

**Authors:** Linda S. Kornerup, Sergey N. Fedosov, Christian B. Juul, Eva Greibe, Christian W. Heegaard, Ebba Nexo

**Affiliations:** 1 Department of Clinical Biochemistry, Aarhus University Hospital, Palle Juul-Jensens Boulevard 99 8200 Aarhus N, Denmark; 2 Department of Molecular Biology and Genetics, Aarhus University, Aarhus, Denmark

**Keywords:** Hydroxocobalamin, Cyanocobalamin, Intestinal absorption, Cobalamin deficiency, Kinetic modelling

## Abstract

**Purpose:**

Hydroxocobalamin (HOCbl) is the dominating Cbl form in food, whereas cyanocobalamin (CNCbl) is common in vitamin pills and oral supplements. This study compares single-dose absorption and distribution of oral HO[^57^Co]Cbl and CN[^57^Co]Cbl in Cbl-deficient and normal rats.

**Methods:**

Male Wistar rats (7 weeks) were fed a 14-day diet with (*n* = 15) or without (*n* = 15) Cbl. We compared the uptakes of HO[^57^Co]Cbl (free or bound to bovine transcobalamin) and free CN[^57^Co]Cbl administered by gastric gavage (*n* = 5 in each diet group). Rats were sacrificed after 24 h. Blood, liver, kidney, brain, heart, spleen, intestines, skeletal muscle, 24-h urine and faeces were collected, and the content of [^57^Co]Cbl was measured. Endogenous Cbl in tissues and plasma was analysed by routine methods.

**Results:**

Mean endogenous plasma-Cbl was sevenfold lower in deficient vs. normal rats (190 vs. 1330 pmol/L, *p* < 0.0001). Cbl depletion increased endogenous Cbl ratios (tissue/plasma = *k*_in_/*k*_out_) in all organs except for the kidney, where the ratio decreased considerably. Twenty-four-hour accumulation of labelled Cbl showed that HOCbl > CNCbl (liver) and CNCbl > HOCbl (brain, muscle and plasma).

**Conclusions:**

The Cbl status of rats and the administered Cbl form influence 24-h Cbl accumulation in tissues and plasma.

**Electronic supplementary material:**

The online version of this article (doi:10.1007/s00394-017-1424-0) contains supplementary material, which is available to authorized users.

## Introduction

Vitamin B12 (cobalamin, Cbl) is essential for a normal neurological function and formation of blood cells. The vitamin is supplied via dietary animal products and increasingly through food fortification or vitamin pills [1]. The Cbl forms present in foods are the coenzymes methyl- and 5′-deoxyadenosyl-Cbl (MeCbl, AdoCbl). A brief exposure to light converts both coenzymes to hydroxo-Cbl (HOCbl), making HOCbl the ubiquitous food form of Cbl [2]. Cyano-Cbl (CNCbl) is chemically stable, and it is the predominant Cbl form used in industrial Cbl products (food fortification and vitamin pills) [3]. The general concept is that HOCbl and CNCbl are comparable concerning their absorption and tissue distribution patterns, while free Cbl is absorbed more efficiently than food bound Cbl (for a review see ref. [3]). Our recent data in normal rats challenge both of these statements [4]. We compared 24-h oral absorption of CN[^57^Co]Cbl (free or bound to bovine transcobalamin (TC), the Cbl binding protein in milk) and free HO[^57^Co]Cbl. We found no difference between TC-bound and free CN[^57^Co]Cbl, as well as no difference in the total absorption levels of the two Cbl forms. However, the liver HO[^57^Co]Cbl accumulation was twice as high as the CN[^57^Co]Cbl accumulation. Notably, this result challenges the concept that CN[^57^Co]Cbl and HO[^57^Co]Cbl behave alike and thereby are of equal value for treatment/prevention of Cbl deficiency.

The current study was undertaken to investigate whether the observed differences in distribution of HOCbl and CNCbl also were mirrored in other tissues than liver and kidney and whether this distribution was dependent on Cbl status. In addition, we wanted to explore whether the food form of Cbl (HOCbl) was absorbed alike when administered free or bound to bovine TC.

## Materials and methods

### Animals

Thirty male Wistar rats (Taconic Bioscience Inc., Denmark) were used for the experiments; 7 weeks old, weighing approx. 200 g upon arrival to the animal facilities. The rats were housed in pairs in standard cages (Makrolon 1291 H type III H, 800 cm^2^, Tecniplast, Italy) with free access to food and tap water. The room temperature was 19–20 °C and the humidity 60% with a 12/12 h light/dark cycle. Bedding material (asp chips, Tapvei, Finland) and soft paper wool (LBS biotech, United Kingdom) were changed daily. Rats were kept for 2 weeks, during which half (*n* = 15) were randomized to a Cbl-deficient diet (Altromin C1024, Brogaarden, Denmark) and the other half (*n* = 15) to the control diet (Altromin C1000, Brogaarden, Denmark). The calorie contents of the two diets were equal, but the Cbl-deficient diet contained less cellulose and corn starch and more sucrose compared with the control diet. The manufacturer assessed Cbl content by using the tabulated values for Cbl in different food sources. Therefore, we quantified Cbl in the diets by extracting 0.3 g of solids with 1.5 mL of water. After centrifugation, Cbl was measured in the supernatant employing a Cobas 6000 (Roche Diagnostics). During the analysis, all Cbl is converted to CNCbl; thus, the Cbl content was calculated employing the molecular weight for CNCbl, MW: 1355. The mean of two independent measures is shown (Cbl-deficient diet: <0.5 (<0.5, <0.5) µg/kg, control diet: 60 (69, 51) µg/kg).

All experiments were conducted in agreement with EU Directive 2010/63/EU on animal experiments.

### Study design and experimental procedures

**Fig. 1:**
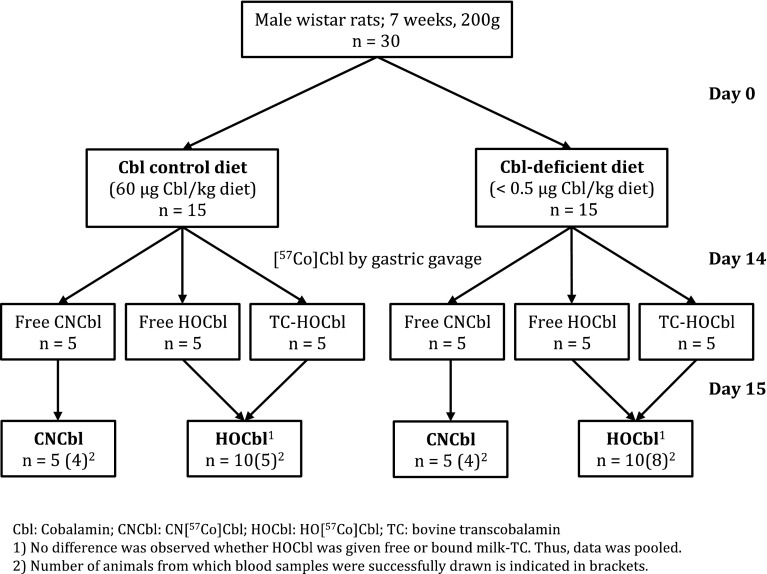
Study design. *Cbl* Cobalamin, *CNCbl* CN[^57^Co]Cbl, *HOCbl* HO[^57^Co]Cbl, *TC* bovine transcobalamin

The study design is depicted in Fig. 1. Each of the three subgroups received approx. 150,000 cpm (0.21 pmol) of free CN[^57^Co]Cbl, free HO[^57^Co]Cbl or HO[^57^Co]Cbl in complex with recombinant bovine TC (all dissolved in a 0.15 mol/L solution of sodium chloride). The exact amount of administered radioactivity (in cpm) was calculated based on measurement of the administered volume and the cpm present in 1 mL of the administered solution.

Twenty-four hours prior to sacrifice, 1 mL of the Cbl solution was administered by gastric gavage. Following the oral Cbl dose, the rats were transferred to separate metabolic cages for 24 h. The rats were anesthetized with isoflurane gas, and blood samples were collected by cardiac puncture into lithium-heparin tubes. Afterwards the rats were sacrificed by cervical dislocation. The liver, kidneys, spleen, heart, small intestine, muscle (thigh), brain as well as 24-h urine and faeces were collected, weighed and stored at −80 °C until further processing. Blood samples were centrifuged (9 min, 1850 g), and plasma was separated and stored at −20 °C until analysis. Blood collection failed in 9 rats (two depleted rats receiving HO[^57^Co]Cbl, one depleted rat receiving CN[^57^Co]Cbl, five normal rats receiving HO[^57^Co]Cbl and one normal rat receiving CN[^57^Co]Cbl).

### Reagents and biochemical methods

**Fig. 2:**
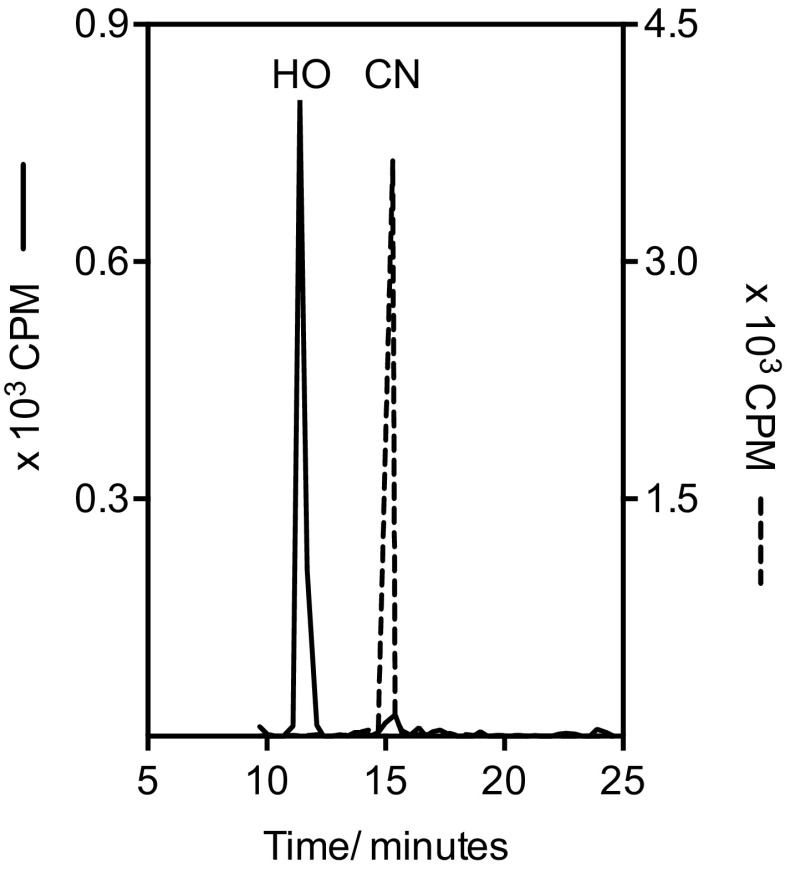
HPLC profile of HO[^57^Co]Cbl after 5-month storage at pH 6.0, 4 °C. Fractions were measured by gammacounter. The result is shown in full drawn line. For comparison, the elution profile of CN[^57^Co]Cbl is shown in *dashed line. HO* HO[^57^Co]Cbl, *CN* CN[^57^Co]Cbl

Commercially available preparations of CN[^57^Co]Cbl (1.75 μCi/mL and 0.41 μCi/pmol Cbl) were used (MP Biomedicals, Ohio, USA, Catalogue no. 06B-430000). Radioactive CN[^57^Co]Cbl was converted into HO[^57^Co]Cbl by photoaquation in an acidic medium under nitrogen bubbling as previously described [4]. The purity of the conversion product was analysed by HPLC employing an in-house method [5] and found to be >95% both at the time of production (data not shown) and after storage under conditions, known to ensure stability of the product (pH 6.0 at 4 °C) [6] for 5 months (Fig. 2).

TC was expressed as previously described [7, 8]. HO[^57^Co]Cbl complexed with TC was prepared by incubating a 10% molar excess of TC with HO[^57^Co]Cbl for 1 h.

[^57^Co]Cbl (cpm) was measured by gamma counter (2470 Wizard^2^ Automatic Gamma Counter, Perkin Elmer, USA).

*For quantification of tissue [* ^*57*^ *Co]Cbl accumulation*, all tissues were thawed on ice, were cut into smaller pieces if necessary and were transferred to tubes for the gamma counter. All tubes were counted to obtain the whole-organ cpm. Intestines and contents were cut and counted together. 24-h faeces and 1 mL urine from each rat were counted. For the calculation of total plasma radioactivity, cpm/mL plasma and estimates of total plasma volume were used [9]. For calculation of the total muscle cpm, cpm/g of muscle and an estimate of the total skeletal muscle mass based on body weight were used [10]. All results were expressed as a fraction of the total administered dose of [^57^Co]Cbl per animal. Additional parameters were employed for analysis of the refined kinetics analysis; see supplementary material.

Six deficient and six normal rats were chosen randomly for the analysis of endogenous Cbl. The samples were thawed on the day of processing. From homogenous tissues, random 250 mg was used (liver, muscle and spleen). From heterogeneous tissues, the apex of the heart, a quarter of a kidney and mixed cerebrum were used (250 mg). We added 750 μL of Na-acetate buffer (0.4 mol/L, pH 4.4) to the individual tissue samples and homogenized at 6800 rpm in three cycles of 20 s with 30 s pauses between cycles (Precellys 24, Bertin Technologies). After homogenisation, 20 μL KCN (30 mmol/L) was added, and the mixtures were boiled (100 °C) for 10 min. Supernatants for quantification of Cbl were collected after centrifugation (40 min; 20,000×*g*) and stored at 4 °C until analysed. The results were expressed as pmol Cbl per g of tissue or whole-organ Cbl (pmol; after multiplying by organ weight). Extraction of endogenous Cbl was >90% as judged from independent experiments, where we added HO[^57^Co]Cbl to the homogenates prior to further treatment, and counted the amount of label remaining in the final supernatant.

To exclude confounding by a difference in biomarkers, we analysed markers of thyroid, liver and kidney function as well as markers of lipid metabolism, as outlined below.

We employed standard laboratory methods for analysing total Cbl (ADVIA Centaur CP immunoassay System, Siemens Healthcare Diagnostics, Denmark), triiodothyronine (T3), thyroxine (T4), alanine aminotransferase (ALAT), total cholesterol, triglyceride, carbamide (Cobas 6000, Roche Diagnostics) and methylmalonic acid (MMA) (6500 QTRAP mass spectrometer, AB Sciex, with a Shimadzu HPLC-system).

### Data analysis and statistical considerations

Our initial analysis showed no difference between rats receiving HO[^57^Co]Cbl (free or bound to bovine TC) and no differences when comparing CN[^57^Co]Cbl with each HO[^57^Co]Cbl group alone, data not shown. Therefore, the two HO[^57^Co]Cbl groups were pooled (Figs. 1, 4). We used GraphPad Prism for Mac OS X, Version 6.0 e, for data analysis. *T* test was used in the comparison of two groups. During analysis of ratios, the mean values of endogenous Cbl (or radioactivity from CN[^57^Co]Cbl or HO[^57^Co]Cbl) in a tissue and plasma were expressed as (tissue Cbl)/(plasma Cbl). The standard error of the obtained value was assessed from the relevant equation for propagation of uncertainties: RSE^2^_*X*/*Y*_ ≈ RSE^2^_*X*_ + RSE^2^_*Y*_ (RSE, relative standard error). All tests were considered statistically significant, when two-tailed *p* values were <0.05.

### Theory of kinetic analysis

**Fig. 3:**
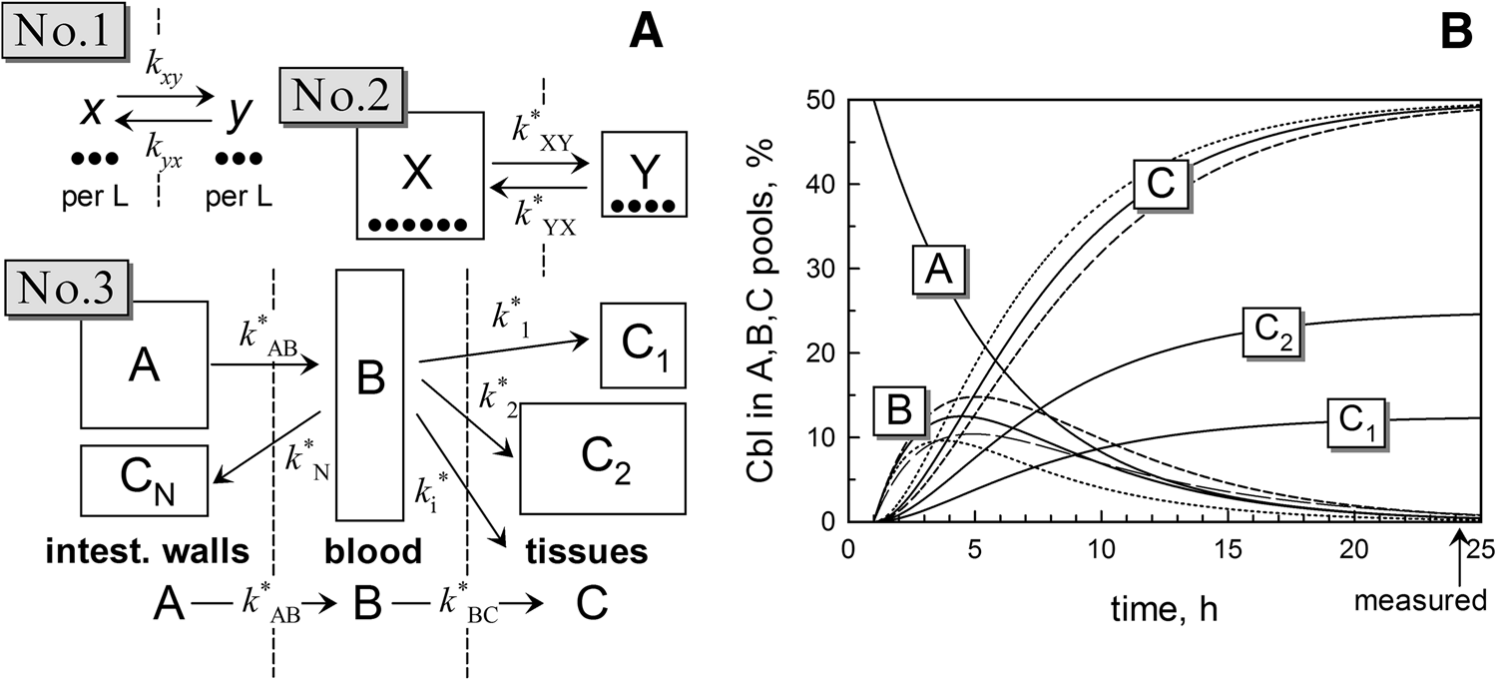
The considered kinetic schemes and simulations. **a** Schemes 1 and 2 show the equilibrium exchange between two compartments and consider the concentration transfer (No. 1) and the total mass transfer (No. 2), respectively. The equal concentrations of *x* and *y* in Scheme 1 (*k*_*xy*_ = *k*_*yx*_) give different masses of *X* and *Y* in Scheme 2 $({k^*}{_{XY} } \ne {k^*}_{XY})$ if the two compartments have different volumes. Scheme 3 describes a unidirectional mass transfer of a metabolite between different compartments (metabolite pools *A, B* and *C*). **b** Simulations of Cbl distribution between the pools *A, B* and *C* (*A*_0_ = 50%). *Solid lines* correspond to the “plausible model” with ${k^*}_{AB} = {\text{ }}0.{\text{2}}\;{{\text{h}}^{ - {\text{1}}}}$ and ${k^*}_{BC} = {\text{ }}0.{\text{4}}\; {{\text{h}}^{ - {\text{1}}}}$. *Curves C*_1_ and *C*_2_ show two examples of Cbl accumulation in the two arbitrary organs 1 and 2 (from the tissue pool *C*) characterized by the mass transfer constants of ${k^*}_{\text{1}} = {\text{ }}0.{\text{1}}\; {{\text{h}}^{ - {\text{1}}}}$ and ${k^*}_{\text{2}} = {\text{ }}0.{\text{2}} \;{{\text{h}}^{ - {\text{1}}}}$, respectively. *Short-dashed curves* show changes in the kinetic records of *B* and *C* after decrease in the overall tissue accumulation constant $\left( {{k^*}_{BC} = {\text{ }}0.{\text{3}}\;{{\text{h}}^{ - {\text{1}}}}} \right)$. *Dotted curves* show the analogous changes at its increase $\left( {{k^*}_{BC} = {\text{ }}0.{\text{6}}\;{{\text{h}}^{ - {\text{1}}}}} \right)$. The *thin long-dashed curve* for the metabolite *B* depicts the time record for blood Cbl at a decreased transfer rate from the intestinal walls to plasma $\left( {{k^*}_{AB} = {\text{ }}0.{\text{15}}\;{{\text{h}}^{ - {\text{1}}}}} \right)$

The following nomenclature was used in the schemes (Fig. 3) and the below equations. Capitalized characters *A, B, C*, etc. denote the mass quantities of cobalamin present in different compartments (e.g. the intestinal walls, blood, tissues) with the volumes (masses) of *V*_*A*_, *V*_*B*_, *V*_*C*_. Lowercase characters *a, b, c*, etc. correspond to the concentrations in the respective compartments. Exchange of Cbl is described by the “true” rate constants (e.g. *k*_*ab*_, *k*_*ba*_ …) for concentrations and the “apparent” rate constants (e.g. ${k^*}_{AB},$ ${k^*}_{BA}$…) for masses. The sequence of subscript characters (e.g. in ${k^*}_{AB}$ or ${k^*}_{BA}$) shows the direction of transfer (e.g. *V*_*A*_ → *V*_*B*_ or *V*_*B*_ → *V*_*A*_).

Figure 3a depicts several examples of the considered kinetic schemes. Scheme 1 shows an equilibrium (e.g. *k*_*xy*_ = *k*_*yx*_ and *x* = *y*), where the law of mass action stipulates the following expression:

1 $${k_{xy}} \cdot x = {k_{yx}} \cdot y.$$

Scheme 2 depicts the identical exchange reaction, where the masses (e.g. *X* = *x*·*V*_X_) are used instead of the concentrations. Here different volumes of compartments (*V*_*X*_ ≠ *V*_*Y*_) cause an uneven distribution of the ligand quantities (*X* ≠ *Y*) despite the equality of concentrations at *k*_*xy*_ = *k*_*yx*_ and *x* = *y*. Such situation requires the new equation of mass balance (Eq. 2).

2 $$k_{XY}^* \cdot X = k_{YX}^* \cdot Y;\;k_{XY}^* = {k_{xy}}\frac{{{V_Y}}}{{{V_X} + {V_Y}}};\;k_{YX}^* = {k_{yx}}\frac{{{V_X}}}{{{V_X} + {V_Y}}}$$

Scheme 3 in Fig. 3a is an approximation that describes a unidirectional Cbl transport from the “excreting compartment” of the intestinal walls (pool *A*) to the blood pool (*B*) and afterwards to different tissues (pool *C* subdivided into *C*_1_, *C*_2_, etc). It should be noted that this approximating model of irreversible transport results in *A* and *B* = 0 at *t* → ∞, and it cannot be applied to an equilibrium. The scheme uses the apparent rate constants dependent on the compartment volumes $({k^*}_j = {k_j} \cdot {V_j}/{\text{S}}{V_j})$. The kinetic equations of Scheme 3 can be presented as follows:

3 $$A = {A_0} \cdot e{^{- k_{A{B^{ - t}}}^*}}$$

4 $$B = {A_0}\frac{{k_{AB}^*}}{{k_{BC}^* - k_{AB}^*}}\left( {{e^{ - K_{A{B^{ - t}}}^*}} - {e^{ - K_{B{C^{ - t}}}^*}}} \right)$$

5 $$C_{i} = A_{0} \frac{{k_{i}^{*} k_{{AB}}^{*} }}{{k_{{BC}}^{*} - k_{{AB}}^{*} }}\left[ {\frac{1}{{k_{{AB}}^{*} }}\left( {1 - e^{{ - k_{{AB^{{ - t}} }}^{*} }} } \right) - \frac{1}{{k_{{BC}}^{*} }}\left( {1 - e^{{ - k_{{BC^{{ - t}} }}^{*} }} } \right)} \right],$$

where *A*_0_ is the amount of Cbl in the first compartment (pool A) at the start of transfer; ${k^*}_{AB}$ and ${k^*}_{BC}$ are the apparent constants of transitions *A → B* and *B → C*, respectively $({\text{where}}\;C\, = \,{\text{S}}{C_i},{k^*}_{BC}\, = \,{\text{S}}{k^*}_i);\,\, {k^*}_i$ is the apparent rate constant of transfer from the pool *B* to a particular compartment *C*_*i*_; *t* is the apparent time of transportation equal to the real time after oral administration of Cbl minus 1 h (*t* = *t*_real_ – 1 h) to start the reactions from Cbl accumulated in the intestinal walls.

The possible curves of Cbl transfer from the intestinal walls *A* to blood and tissues (*A* → *B* → *C*) are presented in Fig. 3b. The shown records illustrate the expected shapes and the amplitudes of Cbl simulated for several sets of ${k^*}_{AB}$ and ${k^*}_{BC}$. The Supplementary materials present more details concerning the theory and the assessment of constants.

## Results

We studied rats fed for 2 weeks on a control diet (60 µg Cbl/kg) or a diet with a reduced Cbl content (<0.5 µg Cbl/kg).

The basic characteristics of the normal and the deficient groups are displayed in Table 1. After 2 weeks on the Cbl-deficient diet, the rats showed biochemical signs of impaired Cbl status. Plasma Cbl values were sevenfold lower and MMA was approximately 30% higher in the deficient group compared with the normal group. The mean body weight of deficient rats was significantly lower (286 g; range 266–298) than that of normal rats (297 g; range 274–318), *p* = 0.02. We found no significant differences in the organ weights (not shown) or in the analysed biomarkers of liver, endocrine or kidney functions (Table 1).

**Table 1:** Basic characteristics for normal and Cbl-deficient rats

	Deficient^a^ *n* = 12	Normal^a^ *n* = 9	*p*
Cbl (pmol/L)	190 (140–300)	1330 (1250–1480)	<0.0001
MMA (μmol/L)	0.88 (0.67–1.15)	0.67 (0.54–0.82)	0.0003
T3 (nmol/L)	1.7 (1.5–2.0)	1.9 (1.7–2.2)	0.06
T4 (nmol/L)	72 (64–84)	74 (62–96)	0.78
ALAT (U/L)	20 (9.0–29)	23 (9.0–35)	0.15
Carbamid (mmol/L)	6.7 (4.6–8.2)	7.0 (5.8–7.9)	0.44
Triglyceride (mmol/L)	1.5 (1.0–2.2)	1.7 (1.0–2.9)	0.48
Total cholesterol (mmol/L)	2.5 (2.1–3.4)	2.3 (1.7–2.9)	0.42

Rats were kept on a control diet (60 µg Cbl/kg) or Cbl-deficient diet (<0.5 µg Cbl/kg) for 2 weeks prior to sacrifice. Results are expressed as mean and (range). *p* = exact *p*-values for comparison of the adjacent columns by two-sided *t* tests

^a^Blood sampling failed in 3 deficient and 6 normal rats

Table 2 displays tissue Cbl content of endogenous Cbl in both normal and deficient rats. The most dramatic differences were found in the kidneys, where Cbl was 93% lower in deficient compared with normal rats. The differences observed in other tissues were less pronounced.

**Table 2:** Tissue contents of endogenous Cbl in normal and Cbl-deficient rats

	Deficient *n* = 6		Normal *n* = 6	
	pmol Cbl/g	Whole-organ Cbl (pmol)	pmol Cbl/g	Whole-organ (pmol)
Liver	20 (18–27)	270 (210–330)	29 (28–30)	370 (325–400)
Kidneys	85 (73–97)	190 (165–225)	1160 (1000–1270)	2400 (2110–2900)
Spleen	10 (9–12)	8 (6–10)	25 (24–29)	20 (15–25)
Heart	28 (27–28)	29 (24–40)	49 (47–51)	51 (47–54)
Brain	13 (12–15)	24 (21–28)	23 (23–24)	45 (43–47)
Muscle	6 (5–8)	635 (465–775)	10 (8–11)	1010 (885–1140)

Rats were kept for 2 weeks on a Cbl-deficient diet or control diet prior to sacrifice. Results are expressed as mean and (range)

**Fig. 4:**
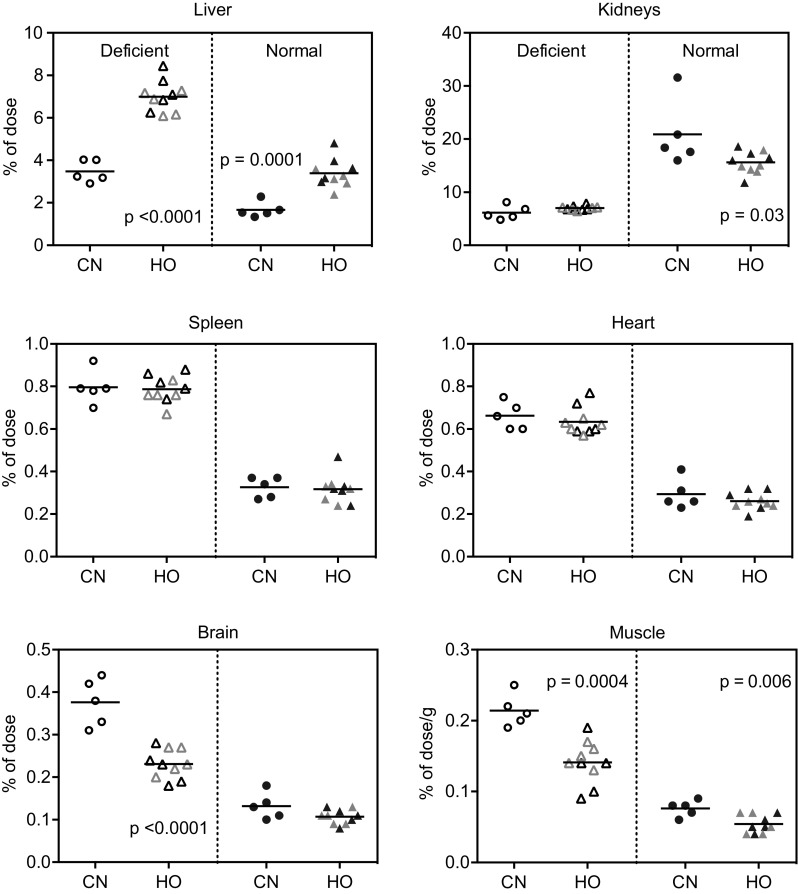
Tissue [^57^Co]Cbl accumulation in normal and deficient rats 24 h after oral administration of CN[^57^Co]Cbl (*n* = 5 in each group) or HO[^57^Co]Cbl (*n* = 10 in each group). Depicted are fractions of the administered [^57^Co]Cbl present in the selected organs per whole organ or per g of muscle. *Horizontal lines* show the mean values. *Scatter symbols* indicate the values for the individual rats. *Filled symbols* normal; *open symbols* deficient; *grey symbols* TC-HO[^57^Co]Cbl. Probabilities of pairwise comparison of the adjacent scatter plots by the *t* test are indicated. *Cbl* Cobalamin, *CNCbl* CN[^57^Co]Cbl, *HOCbl* HO[^57^Co]Cbl, *TC* bovine transcobalamin

**Fig. 5:**
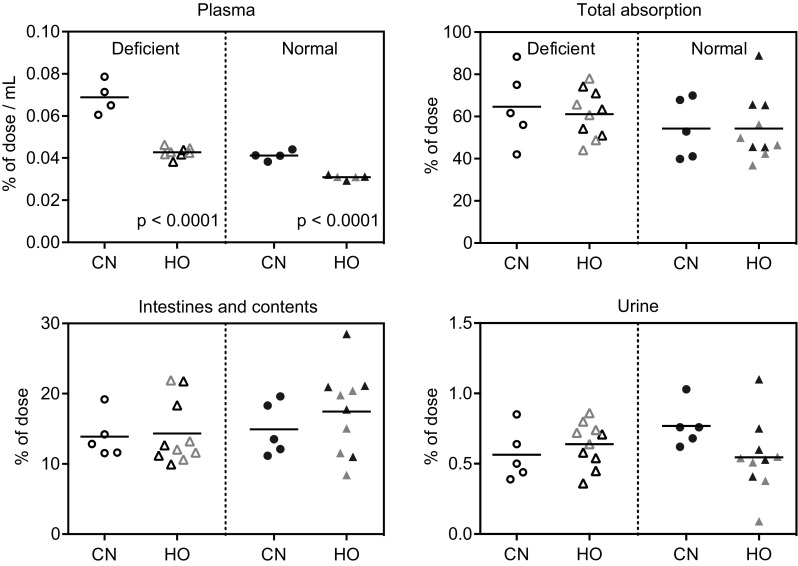
Tissue [^57^Co]Cbl accumulation in normal and deficient rats 24 h after oral administration of CN[^57^Co]Cbl (*n* = 5 in each group) or HO[^57^Co]Cbl (*n* = 10 in each group). Depicted are fractions of administered [^57^Co]Cbl present in plasma (per mL), whole intestines (incl. contents), urine and total absorption. Total absorption = (administered dose minus cpm in ‘intestines and contents’ and faeces)/administered dose. *Horizontal lines* show the mean values. *Scatter symbols* indicate the values for the individual rats. *Filled symbols* normal, *open symbols* deficient, *grey symbols* TC-HO[^57^Co]Cbl. Probabilities of pairwise comparison of the adjacent scatter plots by *t* test are indicated. *Cbl* Cobalamin, *CNCbl* CN[^57^Co]Cbl, *HOCbl* HO[^57^Co]Cbl, *TC* bovine transcobalamin

We studied the 24-h absorption and tissue distribution of orally administered CN[^57^Co]Cbl (free) and HO[^57^Co]Cbl (free or TC-bound). No differences were observed for free HO[^57^Co]Cbl compared with TC-bound HO[^57^Co]Cbl regardless of the Cbl status of the rats (Figs. 4, 5). The results are in agreement with our previous studies for CN[^57^Co]Cbl [4], and we therefore conclude that Cbl (free or bound to TC) is absorbed and distributed alike. Therefore, in our present study, we combined data for uptake of free and TC-bound HO[^57^Co]Cbl. Figures 4 and 5 show the fractions of orally administered [^57^Co]Cbl accumulated in each organ after 24 h. The data for muscle and plasma (% of the administered cpm per g and mL, respectively) should be multiplied by 102 ± 1.3 g and 10.2 ± 0.3 mL (mean ± SD) to give the whole-organ counts. Total absorptions of HO[^57^Co]Cbl and CN[^57^Co]Cbl (equal to the administered dose minus the cpm recovered in intestines and faeces) accounted for approx. 55% of the total administered dose with no significant difference between the groups (Fig. 5).

Distributional differences between the tissues, dependent on both Cbl status and administered [^57^Co]Cbl form, were observed. Notably, deficient rats accumulated threefold less [^57^Co]Cbl in the kidneys, but approximately twofold more in all other organs compared with normal rats. Significantly more HO[^57^Co]Cbl than CN[^57^Co]Cbl accumulated in the liver, while comparable amounts were found in the spleen and heart. Curiously, more CN[^57^Co]Cbl than HO[^57^Co]Cbl entered muscles and brain and remained in plasma.

**Fig. 6:**
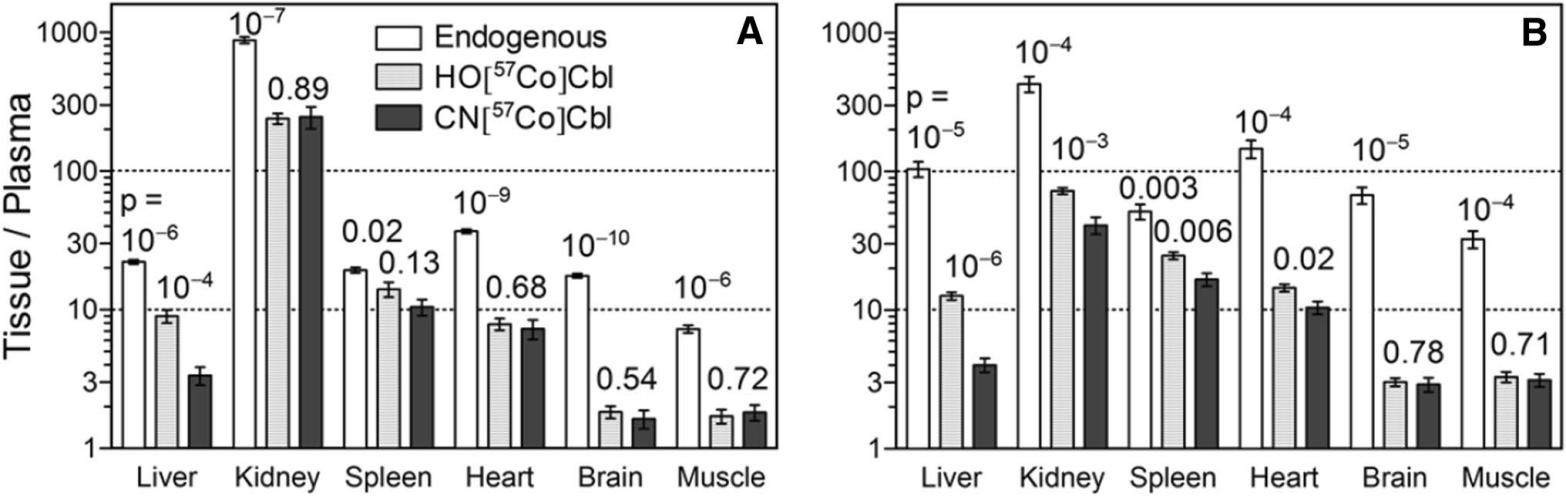
Ratios (mean tissue Cbl/mean plasma Cbl) of endogenous Cbl (pmol/g)/(pmol/mL) (*open bars, n* = 6 normal and, *n* = 6 deficient) and labelled Cbl (cpm/g)/(cpm/mL), accumulated 24 h after administration of oral HO[^57^Co]Cbl (*spotted bars, n* = 5 normal and *n* = 8 deficient) or CN[^57^Co]Cbl (*closed bars, n* = 4 normal and *n* = 4 deficient) in rats kept on a Cbl control (**a**) or a deficient (**b**) diet for 2 weeks. The mean ratio (*bar*) and ± SEM (*vertical* whisker *lines*) are indicated. *Dashed horizontal lines* are depicted to simplify comparison of bars. Probabilities of pairwise comparison of the adjacent bars by *t* test are indicated. *X*-axis indicates the organs examined. *Y*-axis indicates tissue/plasma ratio, log-scale. *Cbl* Cobalamin, *CNCbl* CN[^57^Co]Cbl, *HOCbl* HO[^57^Co]Cbl, *TC* bovine transcobalamin

We also explored the kinetics of [^57^Co]Cbl uptake; the theoretical background and detailed discussion of which may be found in the supplementary material. This points out that the tissue/plasma ratio of [^57^Co]Cbl concentrations is a simple, but illustrative tool to evaluate the basic features of transport kinetics. The ratio for each tissue either reflects its exchange rate constants at the equilibrium (*k*_in_/*k*_out_, plasma ↔ particular tissue) or is proportional to a combination of several forward rate constants (intestine → plasma, plasma → all tissues, plasma → particular tissue) at a transient state. The analysis of ratios for endogenous Cbl (Fig. 6, open bars) shows quite high values and indicates a considerable shift towards Cbl accumulation in all organs of both normal (Fig. 6a) and Cbl-deficient (Fig. 6b) rats. Alignment of normal and deficient rats shows that vitamin deficiency decreases the kidney/plasma Cbl ratio significantly. All other tissues exhibited the opposite tendency, implying that the fractional uptake of circulating Cbl was higher in these tissues. In other words, the deficient rats exhibit a lower in/out transfer balance for the assumed “Cbl depository” kidney, but a higher in/out balance for any “Cbl-utilising” organ when compared with normal rats.

The relatively low values of transient tissue/plasma ratios for [^57^Co]Cbl 24 h after administration (Fig. 6, closed bars, HO/CN[^57^Co]Cbl) indicate that the quasi-equilibrium state was not reached within in 24 h. In normal rats, the spleen ratios are relatively close to the equilibrium values, while particularly the brain and muscle ratios have not reached the equilibrium values. Most tissues exhibit comparable figures for HO[^57^Co]Cbl and CN[^57^Co]Cbl (with a small, yet frequent preference for HO[^57^Co]Cbl). However, in the liver, HO[^57^Co]Cbl uptake is more efficient than CN[^57^Co]Cbl uptake.

## Discussion

We present data on endogenous Cbl and newly absorbed [^57^Co]Cbl in both normal and Cbl-deficient rats. We employed two types of analyses to evaluate the dynamics of the Cbl distribution. First, we assessed [^57^Co]Cbl accumulation in tissues. Second, we estimated the exchange balance between plasma and tissues (expressed via the ratio of mean Cbl concentrations: tissue/plasma). The analyses have some limitations because endogenous Cbl was measured in only six deficient and six normal rats. Plasma values were lacking in 9 animals; thus, the analysis of the transient state was undertaken for 21 of the 30 rats. Yet, we do not consider these limitations to detract from the value of our results since variations observed within each group of rats were small. We used the tissue/plasma ratio as a proxy for the exchange fluxes between the two compartments. This is obviously a simplification of more complex processes, but a reasonable picture can be outlined as long as we stick to a relative (and partially qualitative) assessment of data.

We induced Cbl depletion in rats by keeping them on a Cbl-deficient diet for 2 weeks. Plasma Cbl, MMA measurements and the endogenous Cbl content in kidneys (the Cbl storage organ in rats [11, 12]) confirmed the deficient state. In accordance with previous data [12, 13], Cbl depletion was manifest in all tissues (most pronounced in kidneys). We found 93% lower Cbl levels in deficient kidneys compared with normal kidneys. This value is comparable with the values reported for rats exposed to a deficient diet for up to several months [13–15]. In contrast, the difference in liver Cbl was remarkably smaller. Our rats showed only 30% lower liver Cbl levels in deficient compared with normal rats after 2 weeks. In literature, a more prolonged Cbl restriction led to a 60% decrease after 2 months or 54% after 3 months on the diet [12, 13].

We explored the 24-h absorption and tissue distribution of orally administered HO[^57^Co]Cbl compared with CN[^57^Co]Cbl in both normal and deficient rats. In accordance with our previous data on normal rats [4], we observed comparable absorption levels of the two [^57^Co]Cbl forms in both normal and deficient rats. Interestingly, the tissue distributions of absorbed [^57^Co]Cbl showed noticeable differences between deficient and normal rats as well as between HO[^57^Co]Cbl and CN[^57^Co]Cbl. The liver uptake of HO[^57^Co]Cbl was more than twice that of CN[^57^Co]Cbl, while the brain accumulated more CN[^57^Co]Cbl than HO[^57^Co]Cbl, especially in deficient rats. These results confirm and expand previous findings [4, 16, 17] about higher accumulation of HO[^57^Co]Cbl in the liver and of CN[^57^Co]Cbl in the kidney. Now we show that tissue accumulation patterns apparently fall into three categories: tissues with a preference for HO[^57^Co]Cbl (liver), tissues accumulating comparable amounts of HO[^57^Co]Cbl and CN[^57^Co]Cbl (the spleen and heart; and the kidney of deficient rats), and tissues with a preference for CN[^57^Co]Cbl (brain and muscle; and the kidney of normal rats).

Most previous studies investigated distribution of parenterally administered Cbl, which complicates the comparison of our results with earlier data. However, Adams et al. compared oral absorptions of labelled CNCbl, HOCbl and MeCbl in 63 patients [14]. Administration of 1 μg resulted in mean whole-body retentions of 56% (HOCbl) and 49% (CNCbl) after 16 days, which is comparable to our figures obtained 24 h after Cbl supplementation. Interestingly, these values confirm that HOCbl and CNCbl show a comparable uptake in humans, too.

It should be stressed that higher or lower tissue loads with either one or the other [^57^Co]Cbl form are not a reflection of the individual tissue kinetics, but the result of a combination of parameters for all tissues (see the supplementary material for more details). Presenting this subject in a nutshell: if a ligand *X* vs. *Y* has low ability to enter e.g. the liver, the “remaining” quantity of *X* in the blood will overload all other tissues. In other words, higher uptake levels of CN[^57^Co]Cbl vs. HO[^57^Co]Cbl in, e.g. the muscles and brain (Fig. 4e, f) do not necessarily equal faster CN[^57^Co]Cbl accumulation, but might reflect a slower transport of this ligand into other tissues (e.g. liver). Keeping this in mind, we undertook an analysis of Cbl ratios (tissue/plasma), which provides a more adequate description of the true kinetics of Cbl distribution.

First of all, we observed lower tissue/plasma ratios for newly administered [^57^Co]Cbls (24 h) than for endogenous Cbl in both normal and deficient rats (Fig. 6). We believe that this finding indicates that the equilibrium is not reached, and that [^57^Co]Cbl uptake is still in progress. In the spleen of normal rats, tissue/plasma ratios for newly absorbed [^57^Co]Cbl came closest to the near-equilibrium level, which suggests a rather fast Cbl turnover in this organ (high *k*_in_ and a relatively high *k*_out_). The largest difference in tissue/plasma levels between endogenous and newly absorbed [^57^Co]Cbls was seen in muscle and brain, which probably implies a slower Cbl turnover in these organs.

The observations for plasma Cbl warrant further comments, because in both deficient and normal rats the 24-h plasma samples contain more CN[^57^Co]Cbl than HO[^57^Co]Cbl. Several explanations, as well as combinations of explanations, are possible if we take the time of measurement into account (well after the peak of plasma [^57^Co]Cbl at 4 h for rats [15]). For example, a slower CN[^57^Co]Cbl clearance (plasma → tissues) is a self-suggesting reason, see Fig. 3b (short-dashed modelling curve for *B*-metabolite). Slower CN[^57^Co]Cbl transportation from intestine to plasma is another feasible explanation, because a “tailing” of plasma [^57^Co]Cbl over time is expected in such case (thin, long-dashed curve in Fig. 3b). Finally, a faster backward CN[^57^Co]Cbl excretion from tissues to plasma (i.e. a better retention of HO[^57^Co]Cbl compared with CN[^57^Co]Cbl in the tissues) may be in play. In the latter situation, the bars for “transient” CN[^57^Co]Cbl in Fig. 6 may correspond to a “worsened” near-equilibrium balance (more [^57^Co]Cbl outside) rather than to the decelerated forward transportation. The suggestion of a better HO[^57^Co]Cbl retention could be true if HO[^57^Co]Cbl is more easily converted into the active coenzyme Cbl forms. Regarding this subject, Uchino et al. described a threefold higher conversion of HOCbl than CNCbl to Ado-Cbl in rat liver 24 h after intravenous injection [16]. Other authors found that serum-Cbl increased faster [18, 19] and remained at a higher level for a longer period of time after intramuscular HOCbl injection than after CNCbl injection [18]. Additionally, whole-body retention was higher [20] and urinary excretion lower [19, 21] after HOCbl than after CNCbl injection.

From a biological point of view, it is easy to accept that affinity for the naturally occurring Cbl may be higher than for the synthetic form with a resulting higher accumulation of HO[^57^Co]Cbl in tissues compared with CN[^57^Co]Cbl. In addition, HO[^57^Co]Cbl is much more susceptible to reduction (a necessary step in conversion to the cofactors) than CN[^57^Co]Cbl. It is therefore remarkable that brain and muscle tissue accumulate more CN[^57^Co]Cbl than HO[^57^Co]Cbl in both normal and deficient rats (Fig. 4e, f). Yet, CN[^57^Co]Cbl “overloading” of the brain and muscle may be the direct consequence of “underloading” in other tissues.

Analysis of ratios in Fig. 6 (especially for deficient rats, Fig. 6b) reveals higher ratios for HO[^57^Co]Cbl than for CN[^57^Co]Cbl in a number of tissues (though with different levels of significance). A higher ratio in the non-equilibrium state (Fig. 3a, Scheme 3) requires a faster transportation at one or more forward steps (intestine → plasma; plasma → all tissues; plasma → particular tissue). A higher ratio at the equilibrium (Fig. 3a, Scheme 1) means a shift towards tissue accumulation (plasma ↔ particular tissue). Whatever the reason might be, HO[^57^Co]Cbl appears to be a better choice for faster load into most tissues and/or better internalization percentage. Similar ratio bars for CN[^57^Co]Cbl vs. HO[^57^Co]Cbl for brain and muscle (Fig. 6) apparently indicate a lacking preference for the vitamin form.

[^57^Co]Cbl flux balance (expressed by its tissue/plasma ratio) shows a much lower fractional accumulation in deficient than in normal kidneys, while this parameter increases in all other organs. Usually, rat kidneys accumulate large quantities of Cbl disregarding the small size of this organ. The difference between kidney and other tissues may relate to kidney ultra-filtration and reabsorption of the transcobalamin-Cbl complex by the megalin receptor present in proximal tubules [22]. The kidney has a high capacity to accumulate ultra-filtrated Cbl, which is likely to be directly related to plasma Cbl concentration. Additionally, the Cbl dynamics of the kidney supports its role as a Cbl storage organ. A higher plasma [^57^Co]Cbl in deficient rats will cause a higher kidney [^57^Co]Cbl ultrafiltration. This also implies a higher reabsorption of [^57^Co]Cbl in the proximal tubules of deficient rats since the same level of urine [^57^Co]Cbl was found in the two groups (*p* = 0.94). However, less [^57^Co]Cbl is withheld in deficient kidneys at 24 h, which indicates an increased export from the kidneys to plasma, maintaining a sufficient Cbl supply of other tissues. Kidney [^57^Co]Cbl excretion to urine did not deviate significantly between the two [^57^Co]Cbl forms. In summary, kidney [^57^Co]Cbl accumulation, distribution and excretion into urine change according to the current Cbl status. A regulated Cbl export in kidneys has been suggested in the literature [12], but has, to the best of our knowledge, never previously been reported.

In conclusion, our study demonstrates differences in Cbl distribution dependent on both the current Cbl status and the administered Cbl form. It is well recognized that both forms of Cbl are eventually converted to the two coenzyme forms of Cbl [16], but an earlier clinical study showed that in humans the major part of CNCbl is absorbed without conversion to other forms [23]. Our results warrant a long-term investigation to clarify the differences in tissue distribution of administered HO[^57^Co]Cbl compared with CN[^57^Co]Cbl in order to establish their efficacy for supplementation and treatment.

## Electronic supplementary material

Below is the link to the electronic supplementary material.

Supplementary material 1 (DOCX 59 KB)

Supplementary material 2 (TIF 217 KB)

Supplementary material 3 (TIF 305 KB)

## Abbreviations

AdoCbl: 5′-Deoxyadenosyl-Cbl
ALAT: Alanine aminotransferase
Cbl: Cobalamin/vitamin B12
CNCbl: Cyano-Cbl
HOCbl: Hydroxo-Cbl
MeCbl: Methyl-Cbl
MMA: Methylmalonic acid
TC: Transcobalamin
T4: Thyroxin
T3: Triiodothyronine
