# Supplementary material for: Tissue distribution of oral vitamin B12 is influenced by B12 status and B12 form: an experimental study in rats
Source: Eur J Nutr. 2017 Mar 20;57(4):1459–69. doi: 10.1007/s00394-017-1424-0 (PMC5960002; doi:10.1007/s00394-017-1424-0)
Supplement: Supplementary file 1 — Supplementary material 1 (DOCX 59 KB) [file 394_2017_1424_MOESM1_ESM.docx]

**Tissue distribution of oral vitamin B12 is influenced by B12 status and B12 form.**

**An experimental study in rats.**

LS Kornerup^1^, **SN Fedosov**^2*^, CB Juul^2^, E Greibe^1^, CW Heegaard^2^ and E Nexo^1^

^*^Corresponding author concerning the details of the kinetic analysis

**Introduction**

The supplementary data present the considerations behind the kinetic analysis and provide a detailed account for the results and also a discussion of our analyses. For flow and readability, some overlap with the main text has been allowed.

**Theory**
The following nomenclature was used in the schemes and equations. Capitalised characters *A*, *B*, *C*, etc. denote the mass quantities of cobalamin present in different compartments (e.g. the intestinal walls, blood, tissues). Lowercase characters *a*, *b*, *c*, etc. correspond to the concentrations in the respective compartments. Notations *V_A_, V_B_, V_C_*, etc. represent the total volumes (for blood) or masses (for tissues). Exchange between different compartments is described by the “true” rate constants (e.g. *k_ab_*, *k_ba_* …) for Cbl concentrations and the “apparent” rate constants (e.g. *k^*^_AB_, k^*^_BA_* …) for Cbl masses. The sequence of characters in the subscript to a constant shows the direction of exchange, e.g. *k^*^_AB_* denotes the rate constant of mass transfer from compartment *V_A_* to compartment *V_B_*.

Fig. S1A depicts several examples of the considered kinetic schemes. Scheme 1 illustrates the state of a quasi-equilibrium where the Cbl concentrations (*x* and *y*) in the compartments *V_X_* and *V_Y_* are equal because the true rate constants of the exchange rates are also equal. The law of mass action stipulates the following expression at the equilibrium:

Eq.1

Scheme 2 depicts the identical situation where Cbl quantities (e.g. *X* = *x*·*V*_X_) are used instead of its concentrations. It should be noted that different volumes (*V_X_* and *V_Y_*) cause an uneven distribution of the masses despite equal concentrations per volume unit. Exchange of masses requires substitution of the true rate constants *k_xy_* and *k_yx_* by the apparent constants *k^*^_XY_* and *k^*^_YX_* where the compartment volumes are included. Therefore, the new equation of mass balance at an equilibrium (Eq. 2) substitutes the previous one (Eq. 1).

Eq. 2

Eq. 2 (of the mass balance) can be easily transformed into Eq. 1 (of the concentration balance) by cancelling the identical elements on both sides of Eq. 2.

Scheme 3 in Fig. S1A depicts a transitional state in which Cbl is transported from the “excreting compartment” of the intestinal walls (pool *A*) to the blood pool (*B*) and afterwards to different tissues (pool *C*, further subdivided into *C*_1_, *C*_2_, etc), including the “accumulating compartment” of the intestine. The scheme describes mass transfer and uses the apparent rate constants dependent on the compartment volumes (see an example in Eq. 2). The process is considered to be unidirectional, which is a fair assumption at the small constants of backward transitions and provided reactions have not reached the final equilibrium. The kinetic equations for Cbl mass in each compartment can be stated as follows:

Eq. 3

Eq. 4

Eq. 4A

Eq. 5

Eq. 5A

where *A*_0_ is the amount of Cbl accumulated in the first compartment (pool A) at the start of transfer; *k^*^_AB_* is the apparent constant of *A → B* transition; *k^*^_BC_* is the apparent constant of *B → C* overall transition where all *C*-compartments are pooled (*C* = Σ*C_i_*, *k^*^_BC_* = Σ*k^*^_i_*); *k^*^_i_* is the apparent rate constant of transfer to a particular compartment *C_i_*; *t* is the apparent time of transportation equal to the real time after oral administration of Cbl minus 1 hour (*t* = *t_real_* – 1 h) to start the reactions from Cbl in the intestinal walls; *t_SS_* is the time of transient steady state when *B* does not change (a lag of 1 h is added to *t_SS_*).

At any time of transition, the amount of Cbl accumulated in each particular tissue (*C_i_*, Eq. 5) can be related to the Cbl accumulated in all tissues (*C* = Σ*c_i_*), which gives the ratio of the individual transfer constant (*k^*^_i_*) to the overall constant (*k^*^_BC_*):

Eq. 6

The analogous ratio of *C_i_* (Cbl in a specified tissue) to *B* (Cbl in blood) at an advanced state of reaction (e.g. 24 h) gives a more complex expression, which is proportional to *k_i_^*^* but also depends on other constants:

Eq. 7A

Eq. 7B

Here faster transitions *A*→ *B*, *B* → *C* and *B* → *C_i_* cause the ratio to increase. The same trend is observed when using concentrations and the true constants instead of masses and apparent constants.

Each apparent constant of mass transfer (*k^*^_j_*) can be expressed via the true constant of concentration transfer (*k_j_*) corrected for the tissue volume (mass).

Eq. 8

The opposite transformation is rather simple and has been omitted here.

The possible curves of Cbl transfer from the intestinal walls to blood and tissues (*A* → *B* → *C*) are presented in Fig. S1B, see the Supplementary results section below for more details.

**Supplementary results**

*Construction of the kinetic curves of Cbl uptake*

We made a plausible reconstruction of the Cbl uptake kinetics using a two-step scheme *A* → *B* → *C* (intestinal walls → blood → tissues). The data from the literature were used [1] where CN^57^Cbl was orally administered to rats fed on a standard diet (with adequate Cbl). It was shown that approximately 50% of the dose was accumulated in the intestinal walls 1 h after oral administration of CN^57^Cbl, whereupon no further uptake followed. The time of half clearance from the intestinal walls (*A* →) could be estimated as 3-6 h. The peak of Cbl radioactivity in blood (→ *B* →) was observed at approx. 4 h. Finally, half accumulation in the tissues (→ *C*) was reached at 3-8 h.

The time curves in Fig. S1B were simulated by Eq. 3-5 and present a plausible scenario consistent with the experimental data^1^. The curves start from 1 h after “oral administration” of Cbl (*t* = *t_real_* – 1 h in Eq. 3-5) when 50% of Cbl is already accumulated in the intestinal walls, but its further transfer to blood remains insignificant. The approximate values of rate constants (*k^*^_AB_* = 0.2 h^-1^ and *k^*^_BC_* = 0.4 h^-1^, solid curves) provide the necessary replication of the experimental half-clearance times. It should be noted that a relatively slow transfer of Cbl from the intestinal walls to blood (*A* → *B*) also stipulates slow accumulation of Cbl in all tissues (*C*) disregarding the higher rate constant of the second step (*B* → *C*). The “hidden” fast constant *k^*^_BC_* finds its expression in the position and the amplitude of *B* peak, as well as in the horizontal shifts of the *B*- and *C*-curve. Examples of such shifts are shown in Fig. 2B where changed *k^*^_BC_* is assigned as 0.3 h^-1^ (dashed lines) and as 0.6 h^-1^ (dotted lines). The effect of the slower *A* → *B* transfer (*k^*^_AB_* = 0.15 h^-1^) is illustrated for blood Cbl (*B*) by a thin long dashed line. Uptake in each particular organ follows the same “slow” accumulation pattern independently of the objective rate constant of this process (see example curves *C*_1_ and *C*_2_ in Fig. 2B). The higher or lower value of *k^*^_i_* stipulates, however, a higher or lower amplitude of the final accumulation in the tissue (as explained by Eq. 5A). The deduced plausible coefficients of mass transfer (*k^*^_AB_* = 0.2 h^-1^ and *k^*^_BC_* = 0.4 h^-1^) were used to make a rough assessment of the true uptake constant for each particular organ, see the next section.

*Quantification of Cbl uptake in the tissues*

At the first step, we assessed the Cbl concentration ratios tissue/plasma (equal to *k_bc_*/*k_cb_*, see Eq. 1 and Scheme 1 in Fig. S1A). The obtained values (Fig. 4 of the main text, “equilibrium” bars) were quite high and indicated a considerable shift towards accumulation of Cbl in all organs in both normal (panel A) and Cbl-deficient (panel B) animals. The analogous examination of tissue/plasma ratios for labelled ligands 24 h after administration showed that the equilibrium state was not reached (Fig. 4 of the main text, “transient” bars). This observation substantiates application of the “irreversible” Scheme 3 (Fig. S1A) in the analysis of the transitional state.

The internalised CN[^57^Co]Cbl vs. HO[^57^Co]Cbl in the pooled tissues (including the intestinal walls) amounted to 46% vs. 43% in normal rats and 47% vs. 44% in deficient rats (with an average SD of ± 13%). A similar picture was observed for the total absorption, meaning that the uptake of both Cbls from the gastrointestinal tract is nearly identical. With some reservations, this assumption was extrapolated to the transfer of Cbls from the intestinal walls to blood, see the Supplementary discussion.

Assignment of the same *k^*^_AB_* = 0.2 h^-1^ for both ligands and both types of animals allowed for evaluation of the respective values for *k^*^_BC_* (pooled constant of the Cbl mass transfer from blood to tissues). Nonlinear solution of Eq. 4 with the known values of *k^*^_AB_*, *B* (label in blood) and *A*_0_ (label in blood and all tissues) gave the following values: *k^*^_BC_* = 0.416 h^-1^ (CN[^57^Co]Cbl, normal rats), 0.462 h^-1^ (HO[^57^Co]Cbl, normal rats), 0.337 h^-1^ (CN[^57^Co]Cbl, deficient rats), 0.406 h^-1^ (HO[^57^Co]Cbl, deficient rats). These values should be considered in relation to each other rather than as absolute units because of a possible difference between the blood plasma Cbl (measured) and the total blood Cbl (real).

Evaluation of the mass transfer constant *k^*^_i_* for each tissue was done using Eq. 6 supplied by the known individual tissue count (*C_i_*), the sum of all tissue counts (*C*) and the overall mass transfer constant (*k^*^_BC_*) for each set of data specified by the ligand form and the Cbl status of the animals. However, the interpretation of the results for intestinal walls presents some ambiguity because the tissue count includes both the label being excreted (*A*, assumed to be low) and the newly accumulated label (*C_N_*, assumed to be high), see Fig. 2A. The obtained mass transfer constant for a particular tissue (*k^*^_i_*) was used to calculate the respective concentration transfer constant (*k_i_*) with help of Eq. 8, supplemented by the tissue volume = weight (*V_i_*) and the sum of weights (Σ*V_i_*). The results of these calculations are shown in Fig. S2 where comparison between CN[^57^Co]Cbl and HO[^57^Co]Cbl is done for normal and deficient animals. A clearly faster uptake of HO[^57^Co]Cbl vs. CN[^57^Co]Cbl was detected in the liver at any Cbl status. Vaguely higher accumulation constants were detected for HO^57^Cbl in the kidney (deficient rats), spleen and intestine. Equal or slightly lower uptake constants were found for HO^57^Cbl vs. CN^57^Cbl in the heart, brain and skeletal muscles. The apparent difference between the total tissue counts (see the main text) and the tissue uptake constants in Fig. S2 is scrutinised in the Supplementary discussion section below.

**Supplementary discussion**

We attempted to quantify the pharmacokinetics of CNCbl and HOCbl, relying on the observed facts and some assumptions. Thus, it was found that internalization from the gastrointestinal tract of rats evolves similarly for both Cbl forms, as follows from the overall absorbed radioactivity and the pooled tissue counts. A slightly lower body count of HO[^57^Co]Cbl was attributed to the presence of 2-5% of Cbl analogues in the preparation, and this difference was considered to be insignificant. The scheme of tissue distribution (Fig. S1A) was based on data from the literature^1^ and the currently measured results. We considered a unidirectional scheme of Cbl transfer (Fig. S1A, Scheme 3) because the quasi-equilibrium levels of Cbl (plasma ↔ tissues) were shifted noticeably towards the tissues, and accumulation of the exogenous radioactive ligands was far from its balance.

A plausible imitation of Cbl transitions (Fig. S1B) implies a relatively slow transfer of the ligand from intestinal walls to the blood, followed by a relatively fast step of accumulation in the organs. To avoid misunderstanding, it should be stressed that the tissue accumulation (Fig. S1B, *C*–curve) does not follow its own “fast kinetics”, but proceeds in accordance with the slowest step in the scheme (transition from intestine to plasma, *k^*^_AB_*). Half-times of accumulation are the same for all tissues (disregarding their individual kinetics), whereas the final amplitudes are different if the uptake rate constants are not identical (Fig. S1B, example curves *C*_1_ and *C*_2_). The final level (Eq. 5A) is not solely dependent on the particular rate constant of mass transfer (*k*^*^*_i_*) but relies on its ratio to the sum of all constants (*k^*^_AB_* = Σ*k*^*^*_i_*).

The conceptual difference between the final accumulation level and *k^*^_i_* (or *k_i_*) can be illustrated via the following example with two ligands (*X* and *Y*) and five organs. The first ligand *X* enters all tissues equally well (*k^*^_i_* = 0.1 h^-1^), providing equal fractions of 20% at the end. Another ligand *Y* enters organs №1-№4 with a decreased rate constant (all *k^*^_i_* = 0.05 h^-1^), whereas organ №5 shows no preference (*k^*^*_5_ = 0.1 h^-1^). As a result, the final level of measured *Y* slightly decreases in the tissues №1-№4 (from 20% to 16.7%), but increases noticeably in tissue №5 (i.e. from 20% to 33.3%). Considering the experimental error, the alterations will be considered as “insignificant” for organs №1-№4 (where a 2-fold decrease of the rate constant takes place) but, probably, “significant” for organ №5 (where no change of the rate constant occurred). The ensuing common-sense reasoning suggests an incorrect conclusion about the equal rates of uptake for *X* and *Y* in the organs №1-№4, but a better uptake of *Y* in №5.

For these reasons, the percentages of uptake for CN[^57^Co]Cbl and HO[^57^Co]Cbl in different tissues (the main text, Fig. 3) were recalculated to the individual rate constants using radioactivity in plasma as a scaling variable. Analysis of the concentration transfer coefficients (Fig. S2) shows that most of the tissues are either more receptive to HOCbl or have no preference in choosing CNCbl or HOCbl. Muscles might be an exception because a slightly advantageous CNCbl constant was detected in both cohorts of animals. It should be noted that the size of selections is not sufficient to validate changes on a small magnitude (e.g. ×1.3), and only the liver can be considered as an organ with a documented preference for HOCbl.

Low Cbl status affected the uptake constants leading to a considerably slower entrance of both Cbl forms into kidney, whereas all other tissues (possibly with the exception of intestine) showed the opposite patterns. A probable mechanism of accelerated Cbl uptake in deficient animals may involve over-expression of the specific holoTC receptors in the deficient organs. The decreased kidney uptake may imply hindered filtration of holoTC, but the mechanism of such change is not obvious.

Both normal and deficient animals showed higher levels of CN[^57^Co]Cbl than levels of HO[^57^Co]Cbl in blood 24 h after administration, which is in line with our previous findings [2]. We make the conjecture that a slower clearance of CNCbl from blood (*B* → *C*) is the underlying reason. For example, the dashed *B*–curve in Fig. 2B illustrates a “slow” tissue uptake (*k^*^_BC_* = 0.3 h^-1^) and gives an approximately 2-fold higher Cbl in blood 24 h after oral administration in comparison with the “expected” uptake (*k^*^_BC_* = 0.4 h^-1^, solid *B*–line). The alternative mechanism (within Scheme 3 in Fig. S1A), which may explain the higher plasma CN[^57^Co]Cbl at 24 h, implies its slower transfer from the intestinal walls to the blood (e.g. *k^*^_AB_* = 0.15 h^-1^, thin long dash *B*–curve in Fig. S1B). Yet, equal or lower levels of CN[^57^Co]Cbl inside the intestinal cells (if compared with HO[^57^Co]Cbl) give no strong support to this explanation, at least based on the current data.

Another issue worth discussing is the top level of Cbl in blood plasma (*B*–metabolite) found experimentally and deduced from simulations. The theoretically expected maximal radioactivity in blood corresponds to 10-15% of the total dose (Fig. S1B), if the realistic half-times of accumulation and clearance should be maintained. Yet, the measured top values were considerably lower: 1% at ≈ 4 h for rat [1], 8% at ≈ 8 h for man [3], and 3% at ≈ 7 h for man [4]. Adjustment of the theoretical curve *B* in Fig. 2B to max = 1% is possible (e.g. at *k^*^_BC_* = 15 h^-1^), but this change gives a rather unrealistic positioning of the *B*–curve (not shown) topping at 1.3 h instead of at approximately 4 h. It can therefore be speculated whether a considerable part of Cbl (expected to be within the blood pool) rapidly becomes unavailable for measurements due to either redistribution into a “hidden compartment” or binding to receptors in course of a fast equilibrium *B*(detectable) ↔ *B*(hidden). The two values (“total blood Cbl” = “detectable” + “hidden” and “detectable blood Cbl”) will remain positively correlated to each other at any mechanism. Possible ambiguity in the true levels of Cbl in blood can affect the calculations of absolute values of all rate constants in Fig. S2, whereas their relative estimates (in respect to each other) will remain unchanged.

**Figure legends.**

**Fig. S1.** The considered kinetic schemes and simulations. (**A**) Schemes 1 and 2 show the equilibrium exchange between two compartments and consider the concentration transfer (№ 1) and total mass transfer (№ 2), respectively. The equal concentrations of *x* and *y* in Scheme 1 (*k_xy_ = k_yx_*) give different masses of *X* and *Y* in Scheme 2 (*k^*^_XY_ ≠ k^*^_XY_*) if the two compartments have different volumes. Scheme 3 describes a unidirectional mass transfer of a ligand between different compartments (ligand pools *A*, *B* and *C*). (**B**) Simulations of Cbl distribution between the pools *A*, *B* and *C* (*A*_0_ = 50%). Solid lines correspond to the “plausible model” with *k^*^_AB_* = 0.2 h^-1^ and *k^*^_BC_* = 0.4 h^-1^. Curves *C*_1_ and *C*_2_ show two examples of Cbl accumulation in the two arbitrary organs 1 and 2 (from the tissue pool *C*) characterized by the mass transfer constants of *k^*^*_1_ = 0.1 h^-1^ and *k^*^*_2_ = 0.2 h^-1^, respectively. Dashed curves show changes in the kinetic records of *B* and *C* after decrease in the overall tissue accumulation constant (*k^*^_BC_* = 0.3 h^-1^). Dotted curves show the analogous changes at its increase (*k^*^_BC_* = 0.6 h^-1^). The thin long–dashed curve *B* depicts the time record for blood Cbl at a decreased transfer rate from the intestinal walls to plasma (*k^*^_AB_* = 0.15 h^-1^).

**Fig. S2.** The concentration transfer constants of Cbl (log scale). Bars of HO[^57^Co]Cbl and CN[^57^Co]Cbl (±SEM) represent *k_i_* in different tissues of normal rats (**A**) and in deficient rats (**B**). The calculation procedure is explained in the Supplementary text. The presented values are rather relative than absolute because of the limited kinetic data.

**References**

1. Booth CC, Chanarin I, Anderson BB et al. The site of absorption and tissue distribution of orally administered 56Co-labelled vitamin B12 in the rat. Br. J. Haematol. 1957;3:253–61.

2. Kornerup LS, Juul CB, Fedosov SN et al. Absorption and retention of free and milk protein-bound cyano- and hydroxocobalamins. An experimental study in rats. Biochimie 2015. Available at: <http://linkinghub.elsevier.com/retrieve/pii/S0300908415003818>.

3. Booth CC, Mollin DL. Plasma, tissue and urinary radioactivity after oral administration of 56Co-labelled vitamin B12. Br. J. Haematol. 1956;2:223-36.

4. Carkeet C, Dueker SR, Lango J et al. Human vitamin B12 absorption measurement by accelerator mass spectrometry using specifically labeled (14)C-cobalamin. Proc. Natl. Acad. Sci. USA. 2006;103:5694-9.
