# Supplementary figures and images for: Tissue distribution of oral vitamin B12 is influenced by B12 status and B12 form: an experimental study in rats
Source: Eur J Nutr. 2017 Mar 20;57(4):1459–69. doi: 10.1007/s00394-017-1424-0 (PMC5960002; doi:10.1007/s00394-017-1424-0)

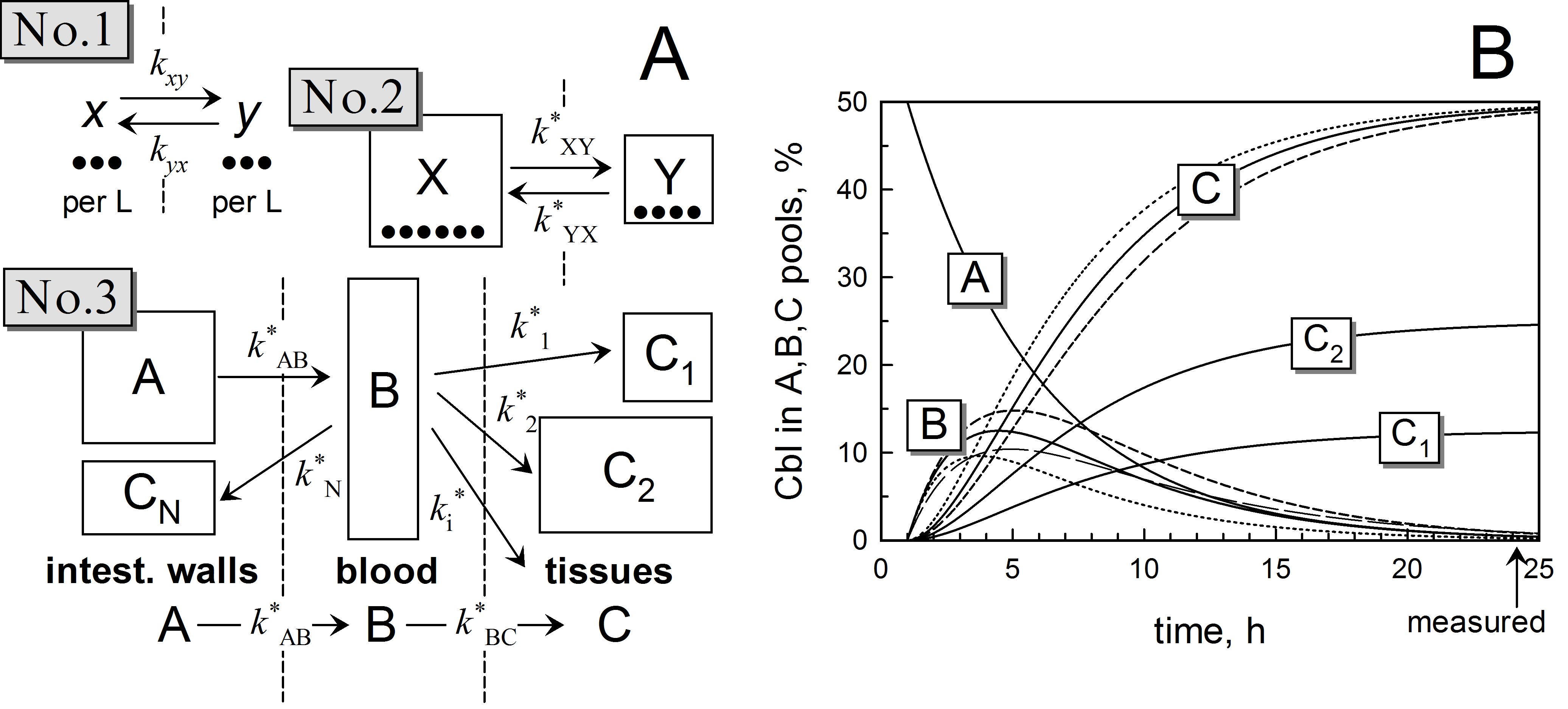

Supplement: Supplementary file 2 — Supplementary material 2 (TIF 217 KB) [file 394_2017_1424_MOESM2_ESM.tif]

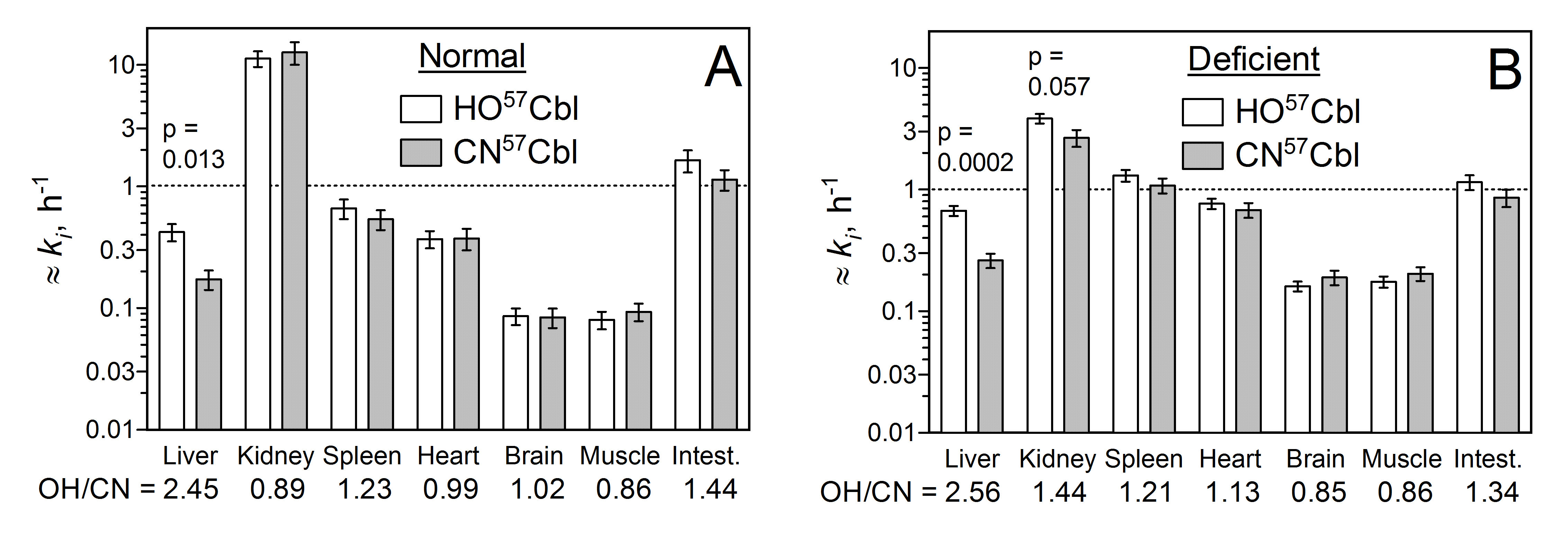

Supplement: Supplementary file 3 — Supplementary material 3 (TIF 305 KB) [file 394_2017_1424_MOESM3_ESM.tif]
